# Cardiovascular Diseases—A Focus on Atherosclerosis, Its Prophylaxis, Complications and Recent Advancements in Therapies

**DOI:** 10.3390/ijms23094695

**Published:** 2022-04-23

**Authors:** Łukasz Bułdak

**Affiliations:** Department of Internal Medicine and Clinical Pharmacology, Medical University of Silesia, Medykow 18, 40-752 Katowice, Poland; lbuldak@sum.edu.pl

Long-term consequences of atherosclerosis remain the major culprit of mortality in developed and developing countries. The pathophysiology of atherosclerosis as an inflammatory disease was explained by Ross [1] in 1989. Since then, there have been major improvements in the prevention, early diagnosis and therapeutic options. However, there are still many challenges to be dealt with. Those include further reduction in mortality, and preventive measures to reduce the complications of acute cardiovascular events and the burden of post-COVID-19 syndrome. Therefore, in this Special Issue of *IJMS,* we focused our attention on the epidemiology, prevention and treatment of cardiovascular diseases, especially in the context of the ongoing COVID-19 pandemic. We have received valuable input from scientists around the world concluding a need for further studies on this subject.

Several recent papers describing the factors responsible for the development of atherosclerosis have been published. According to those finding, gut microbiota might be an essential player in atherosclerosis [2]. Microbes’ metabolites (i.e., bile acids, trimethylamine N-oxide and short chain fatty acids) take part in cholesterol homeostasis, which might suggest their involvement in atherosclerosis. An exploitation of that relationship by modulating gut microbial flora seems to be an interesting therapeutic strategy. Another interesting paper showed the importance of cardiovascular risk profiling in a pediatric population with type 1 diabetes mellitus [3]. Contrary to the general population, it was found that girls are at greater cardiovascular risk compared with boys of a similar age. Lately, we also observed significant increases in viral infections of interest (including SARS-CoV2) that might be responsible for unfavorable cardiovascular outcomes [4].

Within the diagnostic field, in an interesting paper from a research center in Spain [5], some authors devised an in vitro method of the detection of gains and losses of function in the PCSK9 gene that seem to determine whether an easily detectable change in the nucleotide sequence harbors a functional change.

There have also been several papers showing novel treatment options and potential new targets for these therapies. Researchers from South Korea showed promising results of an add-on therapy of ischemic stroke [6]. The intervention was based on the use of otaplimastat and was carried out in an in vivo rat model of ischemic stroke. Otaplimastat was shown to suppress the activity of matrix metalloproteases (MMP) by restoring the level of tissue inhibitor of metalloproteases (TIMP), which resulted in reduced vascular permeation. These data substantiate the potential use of this drug as an add-on therapy to the treatment of ischemic stroke with recombinant tissue plasminogen activator (rtPA). The coadministration might be aimed at prolonging the limited time-period of thrombolytic events and improved clinical outcomes. The abovementioned study gives hope for the treatment of strokes, but efforts should be put predominantly on preventive measures. Vitamin D deficiency has been attributed to several pathologies [7], but an interventional in vivo study in rats published by Sipos et al. [8] showed that vitamin D deficiency is connected with an abnormal response of carotid arteries to stimuli (phenylephrine and acetylcholine), which was associated with a reduction in endothelial nitric oxide synthase levels and increased expression of thromboxane receptors. These alterations are typical for an early phase of atherosclerosis development. Therefore, this is another reason to consider vitamin D supplementation as a valuable option in the prevention of atherosclerosis.

Recently, type 2 sodium-glucose transporter (SGLT-2) inhibitors have been approved for the treatment of congestive heart failure (CHF). The mechanism of action in this indication is not completely clear. An interesting interventional in vivo study in rats by Georg et al. [9] has shed more light on the potential pathways involved in the beneficial effects of SGLT-2 inhibitors. In an experimental setting, some authors observed that low-dose empagliflozin improved cardiac function after myocardial infarction (MI) and reduced MMP-9 expression. Additionally significant improvements were noted in the proteins associated with calcium and sodium transport, notably a reduction in type 1 sodium hydrogen exchanger (NHE1) and an increased expression of sarco/endoplasmic reticulum Ca^2+^-ATPase (SERCA2a). Those mechanisms might be responsible for improved outcomes in heart failure after myocardial infarction. Another drug has also successfully been added to the guidelines of international cardiology societies for heart failure treatment [10]. Sacubitril, belonging to the group of neprilisin inhibitors, is used with valsartan to improve the outcomes of patient with CHF. A major mechanism of action leads to reduced proteolysis of natriuretic peptides, but other pleiotropic effects seem to also be involved. A study in a rat model of MI resulting in mitral regurgitation showed that sacubitril prevented dilation of the left ventricle and reduced fibrosis of the mitral valve apparatus, resulting from reduced transforming growth factor beta expression and diminished endothelial-to-mesenchymal transition [11]. This showed a multifaceted impact of the neprilisin inhibitor on heart remodeling, stretching far beyond a direct impact on natriuretic peptides.

The pathogenesis of atherosclerosis is closely related to other metabolic diseases. For several years, a close connection with non-alcoholic fatty liver disease (NAFLD) has been noted [12]. An interesting therapeutic approach is the therapy of both atherosclerosis and NAFLD with agmatine [13]. Agmatine is a derivative of arginine and was shown to effectively reduce the macrophage content in atherosclerotic plaques, which may reduce the rate of plaque formation and its stability. Furthermore, it improved the triglyceride-to-HDL-C ratio and reduced the triglyceride content in the liver. This compound seems to be a promising therapeutic option, especially in subjects with metabolic syndrome, but currently, further studies are warranted to elucidate the precise mechanism of action that leads to these observed results. Significant metabolic improvements have also been noted during experiments with empagliflozin on hypertrigliceridemic rats [14]. The drug significantly prevented weight gain and reduced fat mass, and the glucose and triglyceride levels with a concurrent rise in HDL-C. Additionally, a decrease in the expressions of enzymes responsible for lipogenesis, markers of inflammatory state and oxidative stress were observed. These observations might be connected to the improved cardio-, hepato- and nephroprotective properties of SGLT-2 inhibitors.

Cell therapy in the treatment of heart diseases has been considered a viable therapeutic option for several years. Its benefits are clear because this therapy might lead to the replacement of lost myocytes. However, there are several obstacles in the common introduction of such a treatment; one is related to the implementation of exogenous cells in the desired fragment of a human body. An interesting approach to more precise engraftment was described by Abizanda et al. [15]. Using a catheter equipped with an irradiation device, the authors were able to selectively apply radiation, which resulted in a significant improvement in the engraftment of ZsGreen^+^ iPSC cells in infarcted rat hearts.

The authors of that manuscript showed further potential pathways and targets for antiatherogenic therapies that might bring about benefits. It seems that there is a plethora of potential options. Gluba-Brzoska et al. [16] described numerous potential targets. The impact on intracellular signal transduction inhibitors (e.g., protein kinase inhibitors and mitogen-activated protein kinases) seems to be of great interest, but confirmatory clinical trials are necessary. Another promising target for the therapy of cardiovascular diseases is vascular endothelial growth factor (VEGF). Several experimental procedures interfering with VEGF signaling have been tested [17]. Currently, approved drugs are used in the treatment of age-related macular degeneration and several types of cancers, but no drug has been approved for the treatment of cardiovascular disease. A wide selection of potential methods of application for blocking intra- and extracellular pathways, including potential therapeutic targets, has been provided by Mahjoubin-Tehran et al. [18]. Further concepts in the modification of the atherosclerotic process involve interference in autophagy [19]. This approach may give rise to novel therapeutic strategies as well as may serve as a diagnostic tool.

In summary, the selection of papers in this Special Issue of *IJMS* clearly show a great interest in the topic of atherosclerosis. Furthermore, the results show novel targets for therapy and hopefully lead to a breakthrough in the therapy of atherosclerosis.

## References

[1] Ross R. (1999). Atherosclerosis—An inflammatory disease. NEJM.

[2] Vourakis M., Mayer G., Rousseau G. (2021). The Role of Gut Microbiota on Cholesterol Metabolism in Atherosclerosis. Int. J. Mol. Sci..

[3] Schweiger D.S., Battelino T., Groselj U. (2021). Sex-Related Differences in Cardiovascular Disease Risk Profile in Children and Adolescents with Type 1 Diabetes. Int. J. Mol. Sci..

[4] Seeherman S., Suzuki Y.J. (2021). Viral Infection and Cardiovascular Disease: Implications for the Molecular Basis of COVID-19 Pathogenesis. Int. J. Mol. Sci..

[5] Uribe K.B., Chemello K., Larrea-Sebal A., Benito-Vicente A., Galicia-Garcia U., Bourane S., Jaafar A.K., Lambert G., Martín C. (2021). A Systematic Approach to Assess the Activity and Classification of PCSK9 Variants. Int. J. Mol. Sci..

[6] Song H.Y., Chung J.-I., Jalin A.M.A., Ju C., Pahk K., Joung C., Lee S., Jin S., Kim B.S., Lee K.S. (2022). The Quinazoline Otaplimastat (SP-8203) Reduces the Hemorrhagic Transformation and Mortality Aggravated after Delayed rtPA-Induced Thrombolysis in Cerebral Ischemia. Int. J. Mol. Sci..

[7] Máčová L., Bičíková M. (2021). Vitamin D: Current Challenges between the Laboratory and Clinical Practice. Nutrients.

[8] Sipos M., Gerszi D., Dalloul H., Bányai B., Sziva R.E., Kollarics R., Magyar P., Török M., Ács N., Szekeres M. (2021). Vitamin D Deficiency and Gender Alter Vasoconstrictor and Vasodilator Reactivity in Rat Carotid Artery. Int. J. Mol. Sci..

[9] Goerg J., Sommerfeld M., Greiner B., Lauer D., Seckin Y., Kulikov A., Ivkin D., Kintscher U., Okovityi S., Kaschina E. (2021). Low-Dose Empagliflozin Improves Systolic Heart Function after Myocardial Infarction in Rats: Regulation of MMP9, NHE1, and SERCA2a. Int. J. Mol. Sci..

[10] Heidenreich P.A., Bozkurt B., Aguilar D., Allen L.A., Byun J.J., Colvin M.M., Deswal A., Drazner M.H., Dunlay S.M., Evers L.R. (2022). 2022 AHA/ACC/HFSA Guideline for the Management of Heart Failure: Executive Summary: A Report of the American College of Cardiology/American Heart Association Joint Committee on Clinical Practice Guidelines [published online ahead of print, 1 April 2022]. Circulation.

[11] Lee S., Hwang H.-S., Song N., Kang G.-H., Choi K.-H., Ji E., Song J.-M., Kang D.-H. (2021). Effect of Neprilysin Inhibition for Ischemic Mitral Regurgitation after Myocardial Injury. Int. J. Mol. Sci..

[12] Gutiérrez-Cuevas J., Santos A., Armendariz-Borunda J. (2021). Pathophysiological Molecular Mechanisms of Obesity: A Link between MAFLD and NASH with Cardiovascular Diseases. Int. J. Mol. Sci..

[13] Wiśniewska A., Stachowicz A., Kuś K., Ulatowska-Białas M., Totoń-Żurańska J., Kiepura A., Stachyra K., Suski M., Gajda M., Jawień J. (2021). Inhibition of Atherosclerosis and Liver Steatosis by Agmatine in Western Diet-Fed apoE-Knockout Mice Is Associated with Decrease in Hepatic De Novo Lipogenesis and Reduction in Plasma Triglyceride/High-Density Lipoprotein Cholesterol Ratio. Int. J. Mol. Sci..

[14] Trnovska J., Svoboda P., Pelantova H., Kuzma M., Kratochvilova H., Kasperova B.J., Dvorakova I., Rosolova K., Malinska H., Huttl M. (2021). Complex Positive Effects of SGLT-2 Inhibitor Empagliflozin in the Liver, Kidney and Adipose Tissue of Hereditary Hypertriglyceridemic Rats: Possible Contribution of Attenuation of Cell Senescence and Oxidative Stress. Int. J. Mol. Sci..

[15] Abizanda G., López-Muneta L., Linares J., Ramos L.I., Baraibar-Churio A., Bobadilla M., Iglesias E., Coppiello G., Ripalda-Cemboráin P., Aranguren X.L. (2021). Local Preirradiation of Infarcted Cardiac Tissue Substantially Enhances Cell Engraftment. Int. J. Mol. Sci..

[16] Gluba-Brzózka A., Franczyk B., Rysz-Górzyńska M., Ławiński J., Rysz J. (2021). Emerging Anti-Atherosclerotic Therapies. Int. J. Mol. Sci..

[17] Dabravolski S.A., Khotina V.A., Omelchenko A.V., Kalmykov V.A., Orekhov A.N. (2022). The Role of the VEGF Family in Atherosclerosis Development and Its Potential as Treatment Targets. Int. J. Mol. Sci..

[18] Mahjoubin-Tehran M., Teng Y., Jalili A., Aghaee-Bakhtiari S.H., Markin A.M., Sahebkar A. (2021). Decoy Technology as a Promising Therapeutic Tool for Atherosclerosis. Int. J. Mol. Sci..

[19] Bu S., Singh K.K. (2021). Epigenetic Regulation of Autophagy in Cardiovascular Pathobiology. Int. J. Mol. Sci..

